# Eltrombopag Added to Standard Immunosuppressive Treatment as Front‐Line Therapy for Severe Aplastic Anemia: Long‐Term Outcomes of the Phase‐3 Randomized Superiority EBMT‐SAAWP RACE Study

**DOI:** 10.1002/ajh.70445

**Published:** 2026-07-19

**Authors:** Antonio M. Risitano, Simona Iacobelli, Austin Kulasekararaj, Marleen van Os, Sofie R. Terwel, Joe Tuffnell, Brian Piepenbroek, Morag Griffin, Constantijn J. M. Halkes, Christian Recher, Fiorenza Barraco, Edouard Forcade, Juan Carlos Vallejo, Beatrice Drexler, Jean‐Baptiste Mear, Roochi Trikha, Shreyans Gandhi, Anna Maria Raiola, L. G. M. Daenen, Marco R. de Groot, Etienne Daguindau, Erfan Nur, Wilma Barcellini, Nigel H. Russell, Louis Terriou, Anna Paola Iori, Walter Barberi, Anna Sureda, Isabel Sánchez‐Ortega, Blanca Xicoy, Isidro Jarque, James Cavenagh, Flore Sicre de Fontbrune, Camilla Frieri, Talha Munir, Jennifer M. L. Tjon, Suzanne Tavitian, Aline Praire, Laurence Clement, Florence Rabian, Luana Marano, Anita Hill, Elena Palmisani, Petra Muus, Serena Marotta, Fabiana Cacace, Marica Laurino, Jakob R. Passweg, Gérard Socié, Ghulam J. Mufti, Carlo Dufour, Régis Peffault de Latour, Severe Aplastic Anaemia Working Party of the EBMT

**Affiliations:** ^1^ Clinical Study Unit EBMT Leiden the Netherlands; ^2^ Department of Clinical Medicine and Surgery Federico II University Naples Italy; ^3^ Hematology and Hematopoietic Transplant Unit AORN S. Giuseppe Moscati Avellino Italy; ^4^ Department of Public Health and Infectious Diseases University of Rome Sapienza Rome Italy; ^5^ Haematological Medicine, King's College Hospital NHS Foundation Trust London UK; ^6^ Leiden Study Unit EBMT Leiden the Netherlands; ^7^ Department of Haematology St James's University Hospital Leeds UK; ^8^ Department of Hematology Leiden University Medical Center Leiden the Netherlands; ^9^ Hematology Department Centre Hospitalier Universitaire de Toulouse Toulouse France; ^10^ Institut Universitaire du Cancer de Toulouse Oncopole Toulouse France; ^11^ Centre Hospitalier Universitaire Lyon Sud Lyon France; ^12^ CHU Bordeaux, Hôpital Haut‐Leveque Pessac France; ^13^ University Hospital of Salamanca Salamanca Spain; ^14^ Hematology University Hospital Basel Basel Switzerland; ^15^ Clinical Hematology, University Hospital of Rennes Rennes France; ^16^ Haematology and Cellular Therapies, IRCCS Ospedale Policlinico San Martino Genova Italy; ^17^ Department of Haematology University Medical Center Utrecht Utrecht the Netherlands; ^18^ Department of Hematology University Medical Center Groningen Groningen the Netherlands; ^19^ Service d'Hématologie, Hopital Jean Minjoz Besancon France; ^20^ Department of Clinical Hematology Amsterdam University Medical Centers, Academic Medical Center Amsterdam the Netherlands; ^21^ UOC Ematologia, UOS Fisiopatologia delle Anemie, Fondazione IRCCS Ca' Granda Ospedale Maggiore Policlinico Milan Italy; ^22^ Centre for Clinical Haematology Nottingham University Hospital Nottingham UK; ^23^ Hôpital Claude Huriez Lille France; ^24^ Department of Translational and Precision Medicine, Division of Hematology Sapienza University Rome Italy; ^25^ Department of Hematology ICO Bellvitge‐Hospital Duran i Reynals L'Hospitalet de Llobregat Spain; ^26^ Executive Office, EBMT Barcelona Spain; ^27^ Institut Català d'Oncologia, Hospital Germans Trias i Pujol Badalona Spain; ^28^ Josep Carreras Leukemia Research Institute Badalona Spain; ^29^ Universitat Autònoma de Barcelona Badalona Spain; ^30^ Servicio de Hematología y Hemoterapia, Hospital Universitario y Politécnico La Fe Valencia Spain; ^31^ Barts Health NHS Trust, St. Bartholomews Hospital London UK; ^32^ French Reference Center for Aplastic Anemia and Paroxysmal Nocturnal Hemoglobinuria, Saint‐Louis Hospital and Université de Paris Paris France; ^33^ Adolescent and Young Adult Hematology Unit Saint Louis Hospital Paris France; ^34^ Hematology Unit IRCCS Istituto Giannina Gaslini Genova Italy; ^35^ Hematopoietic Stem Cells Transplantation and Cellular Therapy Unit AORN Santobono‐Pausilipon Naples Italy; ^36^ Ematologia e Centro Trapianti, IRCCS Ospedale Policlinico San Martino Genova Italy

## Abstract

The RACE study (NCT02009747) compared horse antithymocyte globulin (hATG) plus cyclosporine A (CsA) ± eltrombopag as initial immunosuppressive treatment (IST) for severe aplastic anemia. Here we report the final 2‐year analysis of this prospective randomized phase III study. One hundred ninety‐seven treatment‐naive patients were randomized to standard IST (hATG 40 mg/kg × 4 days and CsA 5 mg/kg/day; arm A; *n* = 101) or standard IST + eltrombopag at the dose of 150 mg/day (arm B; *n* = 96) from day +14 until 6 months (or 3 months, in case of complete response). The median follow‐up was 23.2 months. The 2‐year cumulative incidence of complete response was significantly superior in arm B (62.4% vs. 35.3%; *p* < 0.001). The 2‐year overall survival (OS) was 86% in arm A and 91% in arm B (*p* = 0.081), with hazard ratio (HR), adjusted for age and disease severity, of 0.54 (*p* = 0.064). The 2‐year disease‐free survival (DFS) was 56% vs. 37% (adjusted HR = 0.49; *p* < 0.001), while event‐free survival (EFS) was 48% vs. 32% (*p* < 0.001), with adjusted HR = 0.54 (*p* < 0.001), both significantly superior for arm B. The cumulative incidence of relapse was comparable in the two arms, while evolution to clinical paroxysmal nocturnal hemoglobinuria was 8% in arm A and 1% in arm B (*p* = 0.041). The risk of clonal evolution remained negligible, with one patient in arm A and two in arm B developing karyotypic abnormalities. The initial hematological response benefit of eltrombopag added to IST as front‐line treatment of AA is associated with better 2‐year OS, DFS, and EFS without increased risk of secondary myeloid malignancies.

## 1 Introduction

Acquired immune‐mediated aplastic anemia (AA), previously referred to as idiopathic AA, is the prototype of primary, non‐malignant, bone marrow failure syndrome, which is characterized by a multi‐lineage cytopenia (generally pancytopenia) [1, 2]. The auto‐immune pathophysiology of AA was initially postulated based on clinical observations [3] followed by successful investigations exploiting immunosuppressive treatment (IST) [4, 5], and then confirmed by a number of laboratory studies [6, 7]. Indeed, since the late 1980s IST combining antithymocyte globulin (ATG) with cyclosporine has been the standard of care for AA patients not eligible for hematopoietic stem cell transplantation (HSCT) [8, 9, 10]. After three decades of failed attempts to improve this immunosuppressive regimen [11], the introduction of the thrombopoietin‐receptor agonist eltrombopag, initially in monotherapy for AA patients refractory to IST [12, 13], and subsequently in combination with standard immunosuppression in treatment‐naive patients, resulted in excellent response rates in AA [14, 15].

We conducted a phase III randomized trial to compare standard IST (defined as horse ATG [hATG] plus cyclosporine A [CsA]) with and without eltrombopag [EPAG] as front‐line treatment for patients with AA. The RACE study (NCT02009747) was a prospective, investigator led, open‐label, randomized multicenter trial conducted by the Severe Aplastic Anemia Working Party (SAAWP) of the EBMT enrolling patients diagnosed with severe or very severe AA, who have not received any previous treatment. The analysis of short‐term primary endpoints of this study showed that triple therapy improved rate and quality of hematological response, with higher rates of complete response (CR) at 3 months (21.9% vs. 9.9%, primary endpoint of the study), as well as of overall response rates (ORR) at 3 months and CR and ORR at 6 months, leading to better event‐free survival (EFS) [15]. Although quality and timing of initial hematological response is the best predictor of long‐term survival [8], the course of AA is associated with high risk of relapse (30%–40%), need of re‐treatment, and even risk of progression to myeloid malignancies, the latter accounting for 10%–15% of late failures in historical studies [8, 16, 17]. As a consequence, the impact of eltrombopag as additional therapy associated with IST needs to be investigated in the long‐term, looking for survival and other events which may affect outcome. Here we report the final analysis on the long‐term outcomes of this trial, with all patients completing the 2‐year follow‐up of the study.

## 2 Material and Methods

### 2.1 Study Design

The RACE trial is a prospective, investigator‐led, open‐label, randomized multicenter phase III trial comparing efficacy and safety of hATG + cyclosporine with or without Eltrombopag as front‐line therapy for patients with SAA. The trial was sponsored by the EBMT, and it was conducted at 24 EBMT sites across six European countries [15]. The study was approved by ethical committees of participating institutions, according to European and National regulations. Furthermore, the study was monitored by an independent Data and Safety Monitoring Board. According to the protocol, all registered patients have signed IRB‐approved informed consent, and then were randomly assigned to receive either hATG and cyclosporine alone, or the same regimen, with the addition of eltrombopag. Patient randomization was stratified by age (according to the following two groups, 15–40 or > 40 years), disease severity (severe or very severe), and center. All adverse events were classified according to the Common Terminology Criteria for Adverse Events (CTCAE) [18], and were tracked for the entire 2‐year duration of the study.

### 2.2 Patients

**FIGURE 1:**
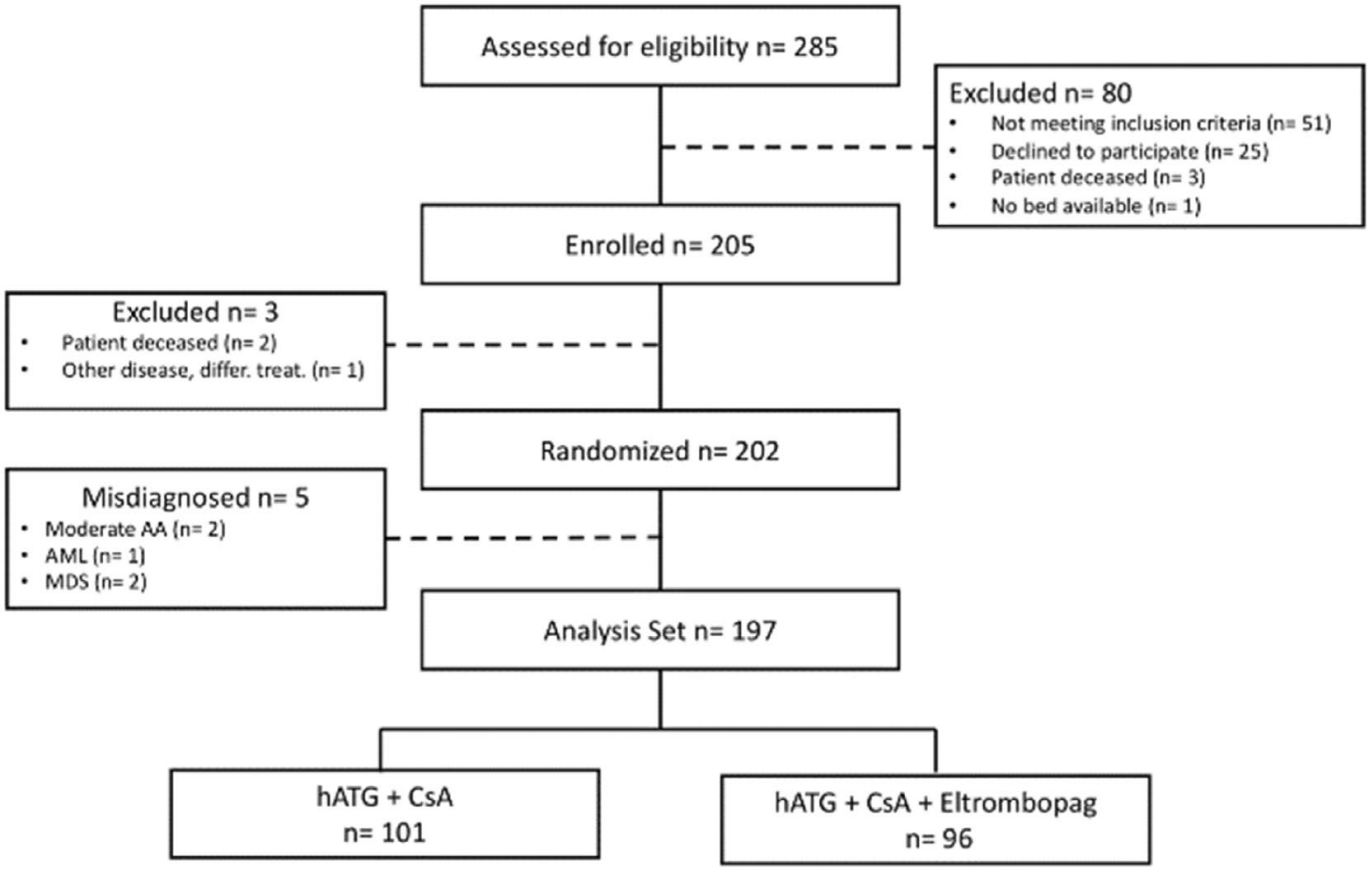
Patients' disposition. From July 2015 to April 2019, 285 consecutive patients aged ≥ 15 years were diagnosed with acquired severe or very severe aplastic anemia at participating sites, and were screened for inclusion in the RACE trial since they were not eligible for front‐line hematopoietic stem cell transplantation. A total of 205 patients were enrolled in the study, and 202 were eventually randomized to receive either standard IST or standard IST + eltrombopag. Five patients were then excluded from the analysis because they were found not to match with inclusion criteria in terms of diagnosis (*n* = 3) or disease severity (*n* = 2).

From July 2015 to April 2019, 285 consecutive patients aged ≥ 15 years with acquired severe or very severe AA who were not eligible for front‐line hematopoietic stem cell transplantation were screened for eligibility in the RACE trial. Two‐hundred and five treatment‐naive patients were enrolled in the trial; three patients were not randomized (two because of early deaths and one due to wrong diagnosis) (Figure 1).

### 2.3 Treatment Protocol

Standard IST (Arm A) consisted of intravenously administered hATG (hATG, ATGAM; Pfizer) at a dose of 40 mg/kg body weight per day on four consecutive days and cyclosporine A, given orally at a dose of 5 mg/kg/day, from day one for a minimum of 12 months and then subsequently tapered in the following 12 months (Appendix S1). Experimental treatment (Arm B) was based on the same standard IST, with the addition of eltrombopag given orally at the dose of 150 mg/d from day +14 until 6 months, except in those patients who achieved complete response (as below defined) at 3 months. Indeed, in Arm B, all patients who were in partial response (as below defined) at 3 months continued eltrombopag to 6 months, per protocol. Still, as per protocol, patients in arm B may also reintroduce eltrombopag beyond 6 months in case of subsequent clinical relapse, or even meaningful drop in blood counts (as defined per protocol). In arm A, patients may also receive eltrombopag beyond 6 months, at the discretion of the treating investigator (as monotherapy, or within a second course of immunosuppressive therapy). In all these circumstances, eltrombopag was considered as additional treatment for AA; other additional treatments include any immunosuppressive drug given outside the treatment regimens (such as second course ATG or alemtuzumab) and androgens.

### 2.4 Endpoints

The study's primary endpoint was hematological complete response (CR) at 3 months as defined by hemoglobin level > 10 g/dL, absolute neutrophil count > 1000/μL, and platelet count > 100 000/μL in the absence of transfusions [10]. Partial response (PR) criteria were transfusion independence (both for red blood cells [RBC] and platelets), with no blood lineage fulfilling the criteria of SAA but insufficient for achieving CR. The short‐term endpoints that have been already reported in the primary report of the study (Table S1) [15]. Long‐term secondary endpoints included overall survival (OS), disease‐free survival (DFS; events: death, no response at 6 months, myeloid malignancy, relapse, transplant), event‐free survival (EFS; events: as for DFS, plus additional AA treatment), relapse (competing events: death, transplant, myeloid malignancy), achievement of first response (overall or CR, competing events: death, transplant, myeloid malignancy, additional AA treatment), evolution to myeloid malignancy and evolution to clinical paroxysmal nocturnal hemoglobinuria (PNH; defined as clinically meaningful hemolysis and/or thromboembolic events) (competing events for both: death and transplant). In line with the definition of competing events for response assessment in the short term, response status at 24 months was defined as No Response if the patient deceased, received additional AA treatment or transplantation, or relapsed, and otherwise based on the actual disease status (CR or PR) at the 24‐months visit. For these latter patients, we additionally reported whether CsA was already discontinued.

### 2.5 Statistical Analysis

The statistical hypothesis justifying the sample size and the statistical methods utilized to compare short‐term response rates (by intent‐to‐treat analysis, with stratification by age, severity and center) was previously described [15]. Here we report the 2‐year final analysis, as pre‐specified in the statistical analysis plan (see supplemental material). As in the previous analysis, all participants with confirmed diagnosis were included and grouped according to the randomization arm. For all time‐to‐event endpoints time started at randomization; relapse and immunosuppression discontinuation curves were left‐truncated at time to first response. Censoring was applied to cases event‐free at last follow‐up. The Mantel–Haenszel (Response), Log‐Rank (survival times) or Gray's tests (endpoints with competing events) to compare treatment arms were stratified on disease severity, age and center. The multi‐variable analysis included disease severity and age as adjustment factors and used robust variance estimation to account for center effect. There were no missing values. The proportional hazards (PH) assumption in Cox models was checked by Scaled Schoenfeld residuals. Where violated, the effect of treatment arm was estimated as piece‐wise constant in three time intervals: 0–6 months, 6–12 months, 12–24 months (time cut points chosen based on background knowledge). Quality of life (QoL) data were collected at times 0, 6, 12 and 24 months using the EORTC QLQ‐30 questionnaire (version 3) and analyzed accordingly. In the present study we focused on the Global health status/QoL (QL2) and on fatigue (FA). For each of them, we presented visualizations based on standard descriptive statistics, and a Linear Mixed Model including the effects of calendar time (as categorical variable, with time 0 as baseline), randomization arm (B vs. A), their interaction, and main baseline characteristics (retained if statistically significant), plus the center effect.

## 3 Results

### 3.1 Patient Disposition

A total of 202 were randomized to receive either standard IST or standard IST + eltrombopag (Figure 1). Five patients were then excluded from the analysis, because they were found not to match with inclusion criteria in terms of diagnosis (*n* = 3) or disease severity (*n* = 2). All remaining patients were followed until death (*n* = 22, 11.1%) or end of study at 24 ± 2 months from randomization, except five patients (2.5%) who withdrew consent to further data collection, and one who anticipated the last visit at month 21 for non‐clinical reasons. For the 170 patients reaching the 2‐year end of study visit the median follow‐up (FU) was 24 months, with 93% having 23‐month FU or longer. All 197 patients were included in the analysis of long‐term outcomes (Table 1).

**TABLE 1:** Patient characteristics at baseline.

Outcome	Total (*N* = 197)	hATG+CsA (Arm A) (*N* = 101)	hATG+CsA + EPAG (Arm B) (*N* = 96)	*p*
Median follow‐up—months (95% CI)	24 (23.9–24.1)	24 (23.9–24.1)	23 (23.9–24.1)	0.60
Median age (min–max)—year	53 (15–81)	52 (15–81)	55 (16–77)	0.43
Age categories—no. (%)				0.44
≥ 15 to < 18 year	9 (4.6)	7 (6.9)	2 (2.1)	
≥ 18 to < 40 year	56 (28.4)	29 (28.7)	27 (28.1)	
≥ 40 to < 65 year	86 (43.7)	43 (42.6)	43 (44.8)	
≥ 65 year	46 (23.4)	22 (21.8)	24 (25.0)	
Sex—no. (%)				0.33
Male	108 (54.8)	52 (51.5)	56 (58.3)	
Female	89 (45.2)	49 (48.5)	40 (41.7)	
Severity of AA—no. (%)				0.80
SAA	129 (65.5)	67 (66.3)	62 (64.6)	
vSAA	68 (34.5)	34 (33.7)	34 (35.4)	
GPI‐deficient neutrophils—no. (%)				0.23
≥ 10%	77 (39.9)	44 (44.0)	33 (35.5)	
Median reticulocyte count (Q1–Q3)—G/L	21 (10–38)	20.0 (8.9–35.8)	23.3 (12.1–46.6)	0.12
Median neutrophil count (Q1–Q3)—G/L	0.4 (0.1–0.8)	0.3 (0.1–0.7)	0.5 (0.1–0.9)	0.08
Median lymphocyte count (Q1–Q3)—G/L	1.4 (1.0–1.8)	1.4 (1.1–1.8)	1.4 (1.0–1.7)	0.44
Median platelet count (Q1–Q3)—G/L	17 (10–30)	18 (10–32)	15 (10–28.2)	0.50
Cytogenetic abnormalities				0.73
Normal—no. (%)	125 (73.5)	64 (74.4)	61 (72.6)	
Abnormal^a^, no. (%)	13 (7.6)	7 (8.1)	6 (7.1)	
Failed, no. (%)	32 (18.8)	15 (17.4)	17 (20.2)	

*Note:* Data set is complete outside the following missing values (total number and percentage, number and percentage in Arm A, number and percentage in Arm B): GPI‐deficient neutrophils (4, 2%; 1, 1%; 3, 3.1%); Reticulocytes (20, 10.2%; 7, 6.9%; 13, 13.5%), Neutrophils (*n* = 4, 2.0%; 2, 2%; 2, 2.1%), Lymphocytes (6, 3%; 2, 2%; 4, 4.2%), Cytogenetic abnormalities (27, 13.7%; 15, 14.9%, 12, 12.5%).

Abbreviations: AA, aplastic anemia; CsA, cyclosporine; EPAG, eltrombopag; GPI, glycophosphatidylinositol; hATG, horse antithymocyte globulin; no., number; PNH, paroxysmal nocturnal hemoglobinuria; Q1, quartile 1; Q3, quartile 3; SAA, severe aplastic anemia; vSAA, very severe aplastic anemia.

^a^ Abnormal karyotype included 7 patients with deletion Y (3 in arm A and 4 in arm B), 2 patients with trisomy 8 in arm A, 1 patient with deletion 20q in arm B, and 3 with other abnormalities.

### 3.2 Hematological Response and Treatment Failures

We previously reported [15] improved CR rates in arm B compared to arm A at 3 and 6 months (Table S1). The superiority of the CR rate was confirmed at 24 months, being 41.9% in arm B vs. 30.3% in arm A, with a persistent statistically significant difference between the two treatment arms (odds ratio = 1.94, 95% CI 1.04–3.63, *p* = 0.037; Figure S1A). This is also shown by the curves of achievement of first CR (Figure S2A), with 24‐month cumulative incidence equal to 62.4% (95% CI, 52.6%–72.2%) in arm B and 35.3% (95% CI, 25.9%–44.7%) in arm A (*p* < 0.001). In multivariable analysis treatment arm A (adjusted hazard ratio (HR) = 1.82; 95% CI, 1.59–2.09, *p* < 0.001), higher age (more than 40 years of age versus 40 years or younger; HR = 0.60; 95% CI, 0.47–0.78, *p* < 0.001) and disease severity (very severe versus severe; HR = 0.69; 95% CI, 0.48–0.98, *p* = 0.036) adversely affected CR achievement. Actual overall response rate (ORR) at 24 months was still superior in arm B (52.7%) versus arm A (36.4%; odds ratio = 2.42, 95% CI 1.31–4.48, *p* = 0.005; Figure S1A and Table S1). The cumulative incidence of achievement of first overall response (Figure S2B) increased in both arms until 12 months; 75.5% (95% CI, 66.9%–84.2%) in arm B and 47.6% (95% CI, 37.9%–57.4%) in arm A (*p* < 0.001). In multivariable analysis treatment arm A (HR = 2.19; 95% CI, 1.64–2.92, *p* < 0.001), higher age (more than 40 years of age versus 40 years or younger; HR = 0.77; 95% CI, 0.60–0.98, *p* = 0.037) and disease severity (very severe versus severe; HR = 0.51; 95% CI, 0.33–0.80, *p* = 0.003) adversely affected response achievement. Reason for treatment failures at 24 months were death and additional, second‐line treatment given for AA (either transplant or non‐transplant options, Figure S1B).

### 3.3 Overall Survival

**FIGURE 2:**
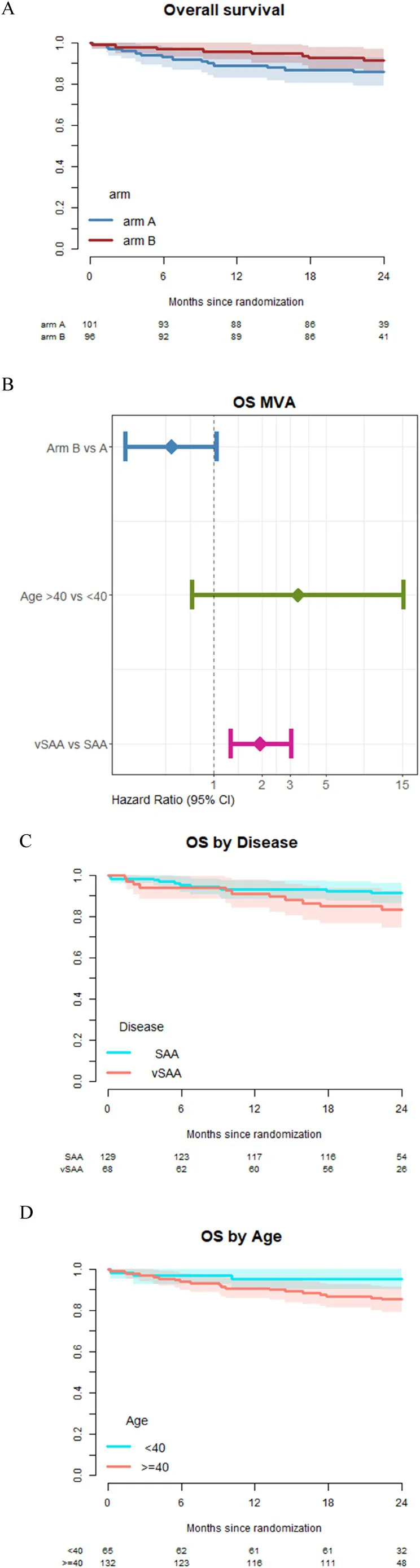
Overall survival (OS). (A) OS Kaplan–Meier curves by arm; (B) OS multi‐variable analysis, forest plot (hazard ratios with 95% confidence intervals); (C) OS Kaplan–Meier curves by disease severity; (D) OS Kaplan–Meier curves by age. [Color figure can be viewed at wileyonlinelibrary.com]

A total of 22 deaths were observed (Table S2), 14 in arm A and 8 in arm B. The most common cause of death was infectious complications (*n* = 13, 59% of all deaths) and bleeding (*n* = 2, 9%). This resulted in a 2‐year OS estimate of 91.4% (95% CI, 85.8%–97.1%) in arm B vs. 86.0% (95% CI, 79.2%–92.8%) in arm A (*p* = 0.081; Figure 2A). In multivariable analysis (Figure 2B), the adjusted HR for arm B compared to arm A was 0.54 (95% CI, 0.28–1.04, *p* = 0.064). Disease severity had a significant impact on OS (HR = 1.95; 95% CI, 1.27–3.01, *p* = 0.002; Figure 2C), while age did not (HR = 3.34; 95% CI, 0.73–15.23, *p* = 0.12; Figure 2D). Explorative comparisons of OS by treatment arms with regard to disease severity and age subgroups are shown in supplemental material (Figure S3). Landmark analysis looking for a possible impact of hematological response did not detect any difference in OS either with respect to overall response (complete and partial) at 3 months or to response at 6 months (Figures S4A and S4B).

### 3.4 Disease‐Free Survival

**FIGURE 3:**
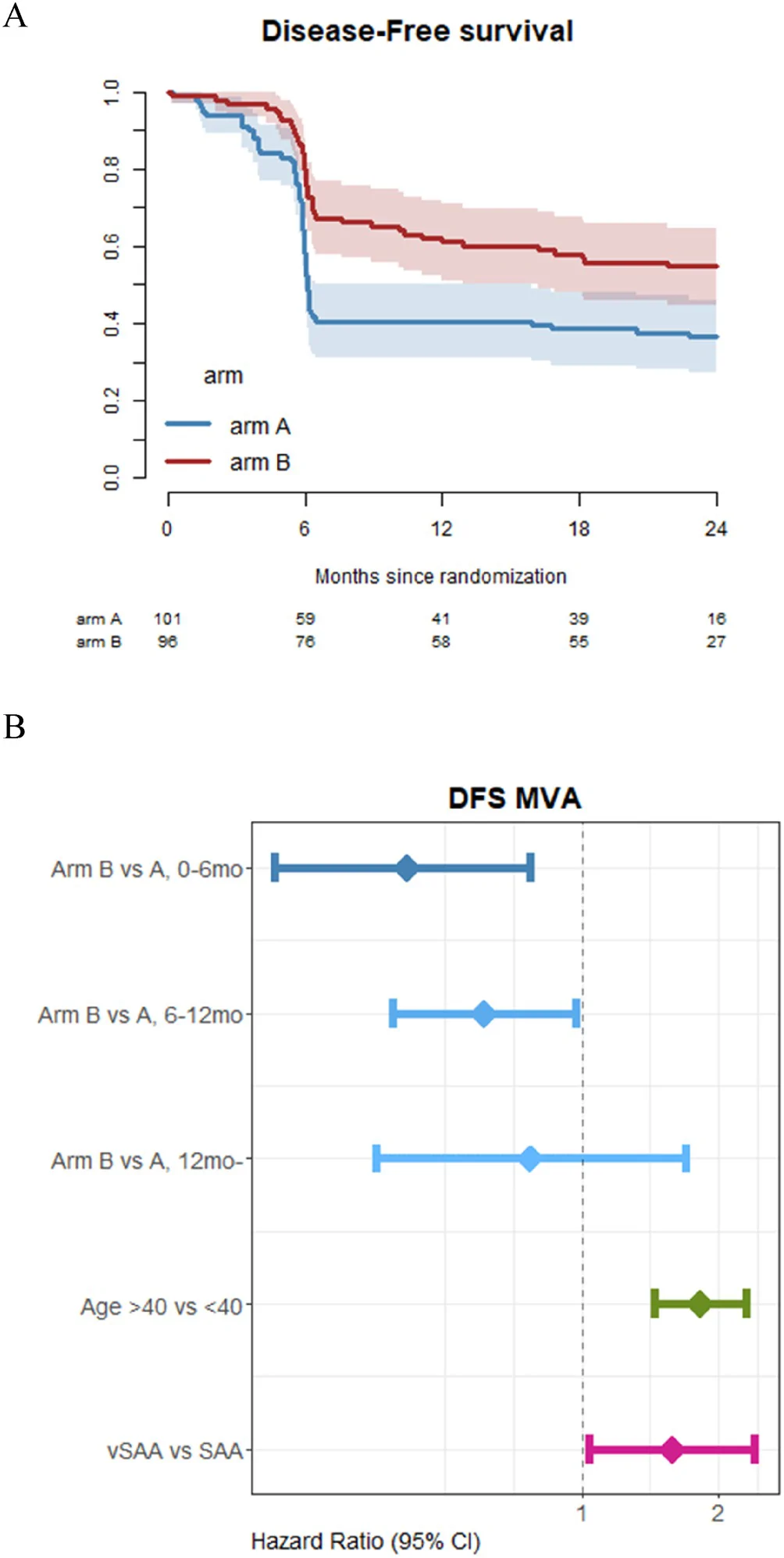
Disease‐free survival (DFS). Events qualifying as failure for DFS were: death, no achievement of response by 6 months, clonal evolution, relapse, transplantation. (A) DFS Kaplan–Meier curves by arm; (B) DFS multi‐variable analysis, forest plot (hazard ratios with 95% confidence intervals). [Color figure can be viewed at wileyonlinelibrary.com]

The 2‐year DFS estimate was 54.7% (95% CI, 44.7%–64.7%) in arm B vs. 36.6% (95% CI, 27.2%–46.0%) in arm A (stratified Log‐Rank test, *p* < 0.001; Figure 3A). In multivariable analysis, treatment arm (HR = 0.50; 95% CI, 0.38–0.66, *p* < 0.001), age (HR = 1.83; 95% CI, 1.44–2.31, *p* < 0.001), and disease severity (HR = 1.59; 95% CI, 1.04–2.43, *p* = 0.034) were found to affect DFS (Figure 3B). Notably, the impact of treatment arm on DFS was time‐dependent, since it was detectable only during the follow‐up intervals 0–6 and 7–12 months (and not 13–24 months).

### 3.5 Event‐Free Survival

**FIGURE 4:**
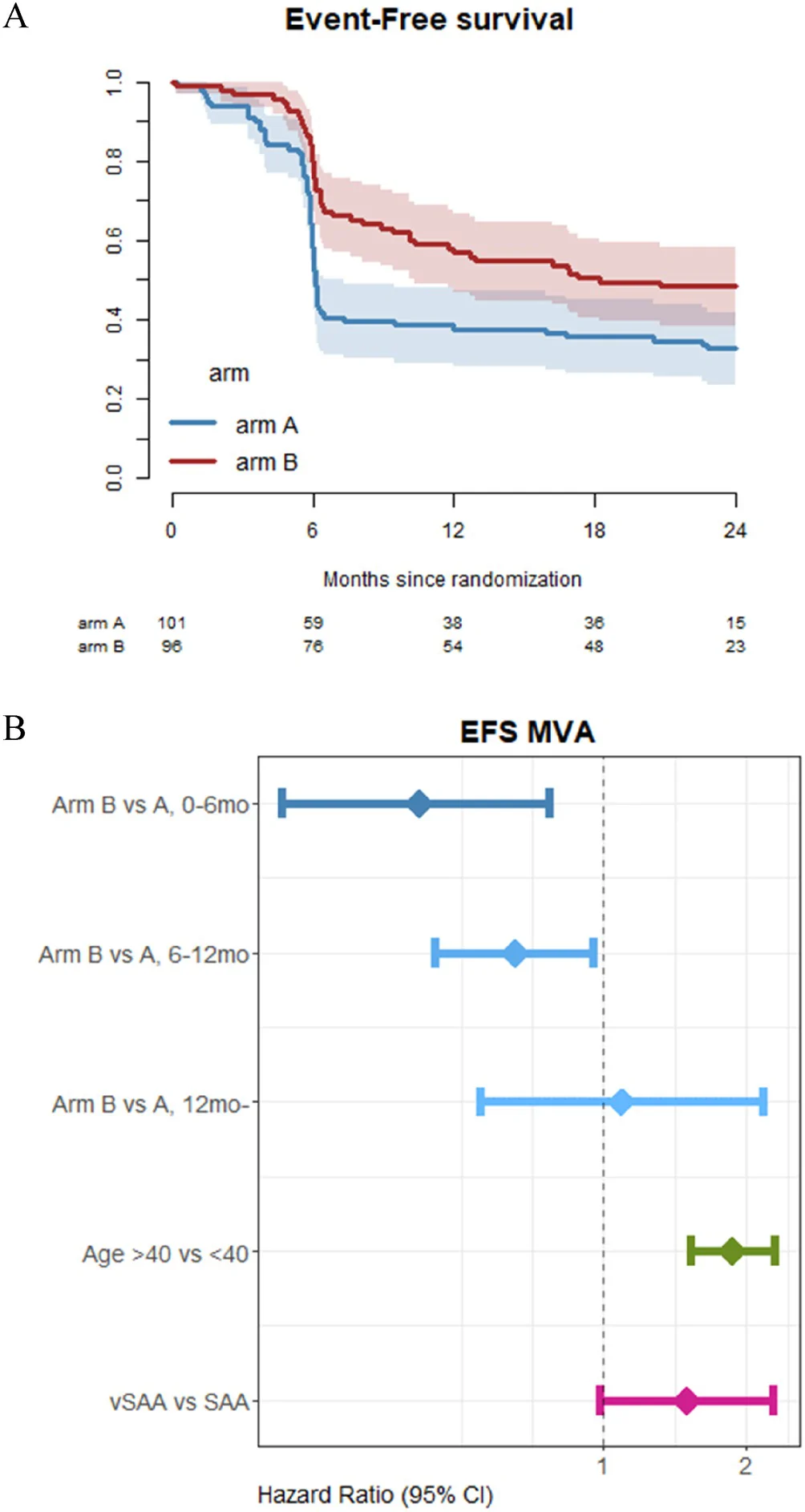
Event‐free survival (EFS). Events qualifying as failure for EFS were: death, no achievement of response by 6mo, clonal evolution, relapse, transplantation, additional AA treatment. (A) EFS Kaplan–Meier curves by arm; (B) EFS multi‐variable analysis, forest plot (hazard ratios with 95% confidence intervals). [Color figure can be viewed at wileyonlinelibrary.com]

When administration of additional treatment for AA was added as an event in the EFS analysis, the 2‐year EFS estimate was 48.4% (95% CI, 38.4%–58.5%) in arm B versus 32.7% (95% CI, 23.5%–41.8%) in arm A (stratified Log‐Rank test, *p* < 0.001; Figure 4A). In multivariable analysis, treatment arm (HR = 0.54; 95% CI, 0.43–0.69, *p* < 0.001) and age (HR = 1.87; 95% CI, 1.51–2.32, *p* < 0.001) but not disease severity (HR = 1.59; 95% CI, 0.98–2.29, *p* = 0.061) were found to affect EFS (Figure 4B). As for DFS, the impact of treatment arm on EFS was time‐dependent, being detected only during the follow‐up intervals 0–6 and 7–12 months (and not 13–24 months).

Looking for the specific events differentially contributing to EFS in the two treatment arms, most of the difference between arms was due to a higher cumulative incidence of lack of response at 6 months in arm A, and to a higher cumulative incidence of additional treatment given in the absence of clinical relapse in arm B (namely eltrombopag re‐introduced per protocol in case of lack of robustness of hematological response; Figure S5).

### 3.6 Safety

Grade 3–5 Adverse Events (AE) occurring during the 2‐year period of the study are listed in the supplemental material (Table S3); no safety concern for the experimental treatment emerged even with long follow‐up. The most frequent grade 3–5 AE were infectious events and hematological events eventually related to the underlying disease, with no differences between the two arms. Severe AE (SAE) were still relatively frequent in this patient population (Table 2): the most common SAE were again infectious events or hematological complications, with no difference between treatment arms. Malignancies remain a rare, albeit possible event, with three cases diagnosed in arm A (a fatal metastatic lung cancer, a metastatic ovarian adenocarcinoma—who died of infectious complications—and a mantle cell lymphoma) and two in arm B (a melanoma and a benign schwannoma—patient died due to complications of cardiac surgery for aortic valve disease) that occur in patients older than 60 years and do not appear related to treatment but to the comorbid status of these patients.

**TABLE 2:** Severe adverse events by arm.

SOC	hATG+CsA (Arm A) (*N* = 101)	hATG+CsA + EPAG (Arm B) (*N* = 96)	Total (*N* = 197)
Blood and lymphatic system disorders	17	17	34
Cardiac disorders	6	4	10
Gastrointestinal disorders	2	15	17
General disorders and admin. site conditions	10	19	29
Hepatobiliary disorders	4	3	7
Immune system disorders	2	5	7
Infections and infestations	53	44	97
Injury, poisoning and procedural complications	1	2	3
Investigations	2	1	3
Metabolism and nutrition disorders	4	2	6
Musculoskeletal and connective tissue disorders	4	1	5
Neoplasms benign, malignant and unspecified	3	2	5
Nervous system disorders	5	5	10
Psychiatric disorders	2	2	4
Renal and urinary disorders	9	11	20
Respiratory, thoracic and mediastinal disorders	8	6	14
Skin and subcutaneous tissue disorders	1	1	2
Surgical and medical procedures	0	1	1
Vascular disorders	2	4	6
Total	135	145	280

Abbreviations: CsA, cyclosporine; EPAG, eltrombopag; hATG, horse antithymocyte globulin; *N*, number; SOC, sistem organ class, as defined by the Common Terminology Criteria for Adverse Events.

### 3.7 Evolution to Myeloid Malignancy

At 2‐year follow up, occurrence of myeloid malignancies (defined as acute myeloid leukemia or myelodysplastic syndrome, according to WHO 2016 criteria) was documented in three patients who developed karyotypic abnormalities (Table S4): one in arm A (a‐7 deletion) and two in arm B (both with 13q‐ deletion). All three patients were alive at the 2 year follow up: the one with 7‐underwent allogeneic transplantation, while the two with 13q‐deletion were in PR and CR (this latter with spontaneous disappearance of the 13q‐clone). No overt AML occurred in both arms.

### 3.8 Evolution to Clinical PNH

**FIGURE 5:**
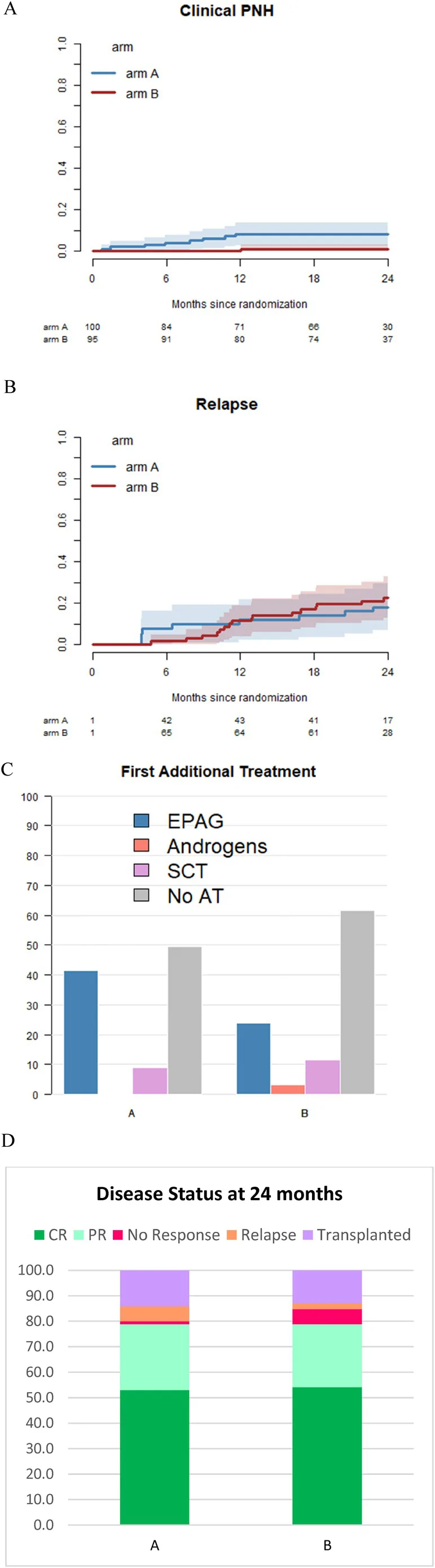
Other secondary endpoints. (A) Cumulative incidence of clinical PNH by arm; (B) Cumulative incidence of relapse by arm; (C) Administration of additional AA treatment by arm (proportions). (D) Disease response status observed at 2 years irrespective of competing failures, by arm; stacked proportions The curves for relapse incidence (panel B) were estimated conditioning on the achievement of response (applying left‐truncation); the number of patients at risk increases when patients achieve response for the first time (entering the risk set) and decreases when patients fail for relapse or for a competing event. [Color figure can be viewed at wileyonlinelibrary.com]

Nine patients developed clinically significant hemolytic PNH during the follow‐up, requiring specific treatment with complement inhibitors. The 2‐year cumulative incidence of clinical PNH was 1.1% (95% CI, 0%–3.2%) in arm B vs. 8.1% (95% CI, 2.7%–13.5%) in arm A (stratified Log‐Rank test, *p* = 0.039; Figure 5A). In multivariable analysis, treatment arm was found associated with the development of clinical PNH (adjusted HR = 0.12, 95% CI, 0.04–0.33, *p* < 0.001), while neither age (HR = 0.70; 95% CI, 0.24–2.06, *p* = 0.518) nor disease severity (HR = 1.14; 95% CI, 0.90–1.44, *p* = 0.265) were found to affect the incidence of clinical PNH (Figure S6A).

### 3.9 Relapse

As expected, relapse was a relatively frequent long‐term event. The 2‐year estimate of cumulative incidence of relapse was comparable between the treatment arms (22.7% (95% CI, 12.9%–32.6%) in arm B versus 18.0% (95% CI, 6.7%–29.3%) in arm A, stratified Gray's test, *p* = 0.82; Figure 5B). In multivariable analysis, age (HR = 4.67; 95% CI, 2.51–8.70, *p* < 0.001) but not treatment arm (HR = 1.05; 95% CI, 0.87–1.27, *p* = 0.603) nor disease severity (HR = 1.16; 95% CI, 0.75–1.79, *p* = 0.509) appeared to affect relapse (Figure S6B). When the cumulative incidence of relapse was estimated from first response, again we did not observe statistically significant differences (Figure S6C). In relapsing patients, the median time to relapse from randomization was 11.9 versus 12.1 months in arm A and B, while the median time to relapse from first response (corresponding to the duration of response) was 4.3 versus 8.0 months, respectively.

### 3.10 Cyclosporine Discontinuation

Per protocol, patients enrolled in both arms received maintenance IST for at least 12 months, followed by slow tapering within the subsequent 12 months, until possible discontinuation around 24 months. At the last follow up study visit, the estimates of responding patients who already discontinued cyclosporine were 25.3% in arm A and 35.1% in arm B (Figure S7); additional patients may have discontinued just after the last study visit. Actual cyclosporine‐dependency could not be assessed at this time point and will be investigated in subsequent long‐term follow up analysis.

### 3.11 Additional Treatments and Disease Status

Due to lack of initial response or subsequent relapses, some patients required additional treatments for their diseases within the 24‐month period of this follow up analysis. Indeed, the proportion of patients who have received any additional treatment was 38.5% in arm B versus 50.5% in arm A; the proportion of patients who have received a minimum of two additional treatments was 5.2% in arm B and 10.9% in arm A (Figure S8). The most common additional treatments were eltrombopag (24.0% in arm B vs. 41.6% in arm A), transplantation (11.5% in arm B vs. 8.9% in arm A), while second course of intensive IST and androgens remained very uncommon (Figure 5C).

When we looked for disease status resulting from these additional treatments (i.e., hematological response status not considering competing events), the rates of CR and PR were 53% and 26% in arm A and 54% and 25% in arm B (*p* = 0.41; Figure 5D).

### 3.12 Quality of Life

**FIGURE 6:**
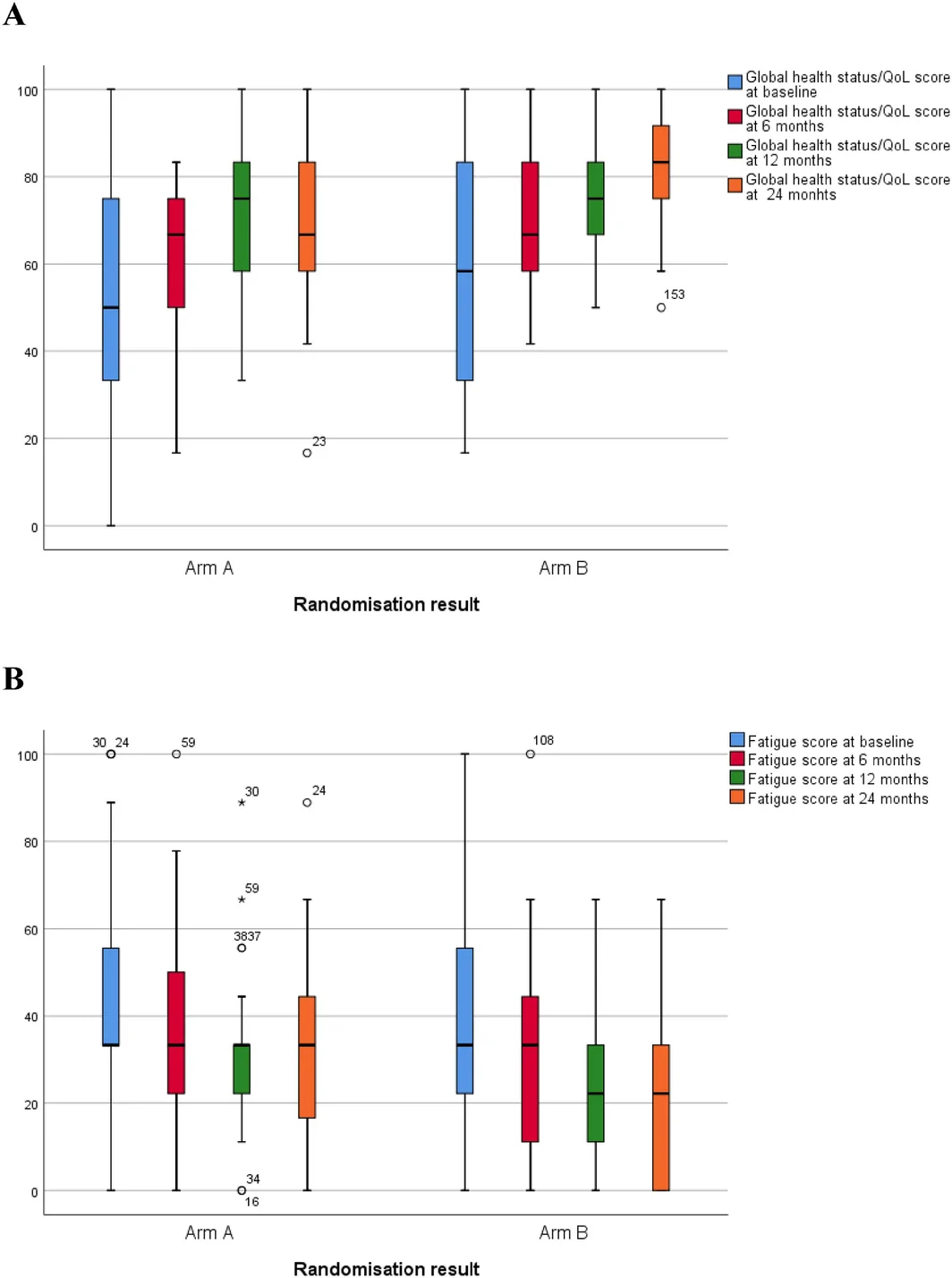
Quality of life. (A) Global health status/QoL (QL2, higher = better); (B) Fatigue (FA, Higher = worse). Boxplots (quartiles). [Color figure can be viewed at wileyonlinelibrary.com]

At baseline, QL2 and FA scores were below and above the normal range reported for healthy individuals, with no statistically significant difference between the two arms. Over time, we observed clinically meaningful improvement of both QL2 and FA in both treatment arms, as illustrated in Figure 6. These trends were confirmed using adjusted mixed models (Table S5 and Figure S9): on both scales QoL improved from baseline over time, with no difference between arms.

## 4 Discussion

Here we report the final 2‐year results of the RACE trial, showing improved rate, quality, and speed of hematological response and improved 2‐year OS, DFS, and EFS with the addition of eltrombopag to standard IST. This improved long‐term outcome was achieved in the absence of increased risk of secondary myeloid malignancies (albeit these are very late events, and the current follow up is still limited) and of any new safety concern, demonstrating the benefit of eltrombopag in this setting. Even with this longer follow up, frequency and severity of adverse events was not different between treatment arms, with excellent tolerance of eltrombopag. Of special note, the difference between the two treatment arms in terms of DFS and EFS largely results from the initial 12 months of treatment (when the exposure to eltrombopag occurs), and it is maintained through later follow‐up, with no significant increased risk of relapse. This observation suggests that eltrombopag is more than a supportive treatment just transiently sustaining blood counts (as G‐CSF) [9, 17], and may rather probably counteract AA pathophysiology restoring normal hematopoiesis. The data confirm the previous phase 2 results reported by the National Institutes of Health (NIH) group, with 6 months CR rates exceeding 30% in both studies [15]. However, some differences in overall response rate are observed, only due to more restrictive criteria for PR in our study which include transfusion independence, and blood counts above the threshold for the definition of SAA. Overall response rate is thus around 80% in both studies when the NIH criteria are applied [14, 15]. We are also reporting a lower relapse rate (about 20%, as compared with the about 40% reported by the NIH group) [19], which is likely due to the shorter follow up (2 vs. 4 years) rather than to the extended cyclosporine maintenance treatment (2 years in our study). This relatively short follow up does also justify for the extremely low rate of clonal evolution to myeloid malignancies, as well as for the excellent overall survival rate, which at 2 years exceeds 90% like in the NIH data [14, 15]. Notably, in our study we could not observe statistically significant difference between the treatment arms in overall survival, as well as in the improvement of quality of life measurements. The former observation is likely explained by effective rescue treatments in the control arm; indeed, the proportion of patients in hematological response at the 2 year follow up was 79% in both study arms. Possibly these excellent long‐term outcomes are also explained by the reduced mortality (especially infectious one) in patients initially not achieving hematological response (as previously published) [20], which then may benefit from additional treatments.

The actual mechanism of action of eltrombopag in AA has not been fully elucidated; both direct effect on hematopoietic multipotent progenitors [21] as well as possible protection from the immune‐mediated inflammatory damage on haematopoiesis [22] were demonstrated. The effectiveness of eltrombopag in AA seems independent from the delivery of an intensive, ATG‐based, immunosuppressive regimen, since (in addition to the initial data on monotherapy, that were achieved in refractory/relapsed patients who have received ATG previously) eltrombopag resulted in meaningful overall hematological response rates even in association with cyclosporine only [23]. This is also confirmed by our observation that around a quarter of patients initially treated with triple therapy eventually required reintroduction of eltrombopag during the follow‐up period to sustain blood counts and prevent clinical relapse. Interestingly, in relapsing patients, the time to relapse was not different among the two treatment arms, while the duration of response tended to be longer in arm B (as consequence of earlier responses). In our study the use of eltrombopag beyond 6 months was permitted in both treatment arms, potentially affecting the difference in OS between the study arms. However, eltrombopag beyond 6 months was more frequently used in the experimental (B) arm. But even when the use of eltrombopag was classified as an event (as in EFS), the long‐term outcome remained largely superior in the eltrombopag arm. Nevertheless, this observation introduces the concept that for some patients longer exposure to eltrombopag may be required, eventually highlighting the fact that the best strategy of discontinuation (*ex abrupto* as per protocol, vs. tapering) is yet to be identified in the daily practice. Furthermore, considering the favorable safety profile of eltrombopag even with long‐term exposure [19], one may raise the question whether in selected patients a chronic treatment with eltrombopag alongside or instead of cyclosporine may be considered, especially due to the nephrotoxicity of the latter agent [24]. In line with this concept, our preference now is to taper eltrombopag (half‐dose every 3 months, or 50 mg every 2 months) before discontinuing it; and some patients extended eltrombopag treatment for the entire duration of the study (i.e., 24 months), akin to cyclosporine.

Our data confirm that eltrombopag in combination with ATG and cyclosporine (the so‐called triple therapy) should be the preferred non‐transplant treatment for adult patients with AA who lack a HLA‐matched sibling donor and for all patients older than 40 years old [2, 25]. This scenario has been recently challenged by the emerging data on upfront haplo‐identical hematopoietic stem cell transplantation, with a 3‐year overall survival rate higher than 90% exploiting a conditioning regimen which includes low/moderate‐dose total body irradiation and ATG associated with post‐transplant high‐dose cyclophosphamide [26]. These very promising data led some expert panels to recommend *considering* [27] or even *prioritizing* [28] haplo‐identical transplant as an initial treatment strategy for a broad population of aplastic anemia patients, even > 40 years old. We might agree that this approach is preferred in children (considering the lack of benefit of add‐on eltrombopag in this setting) [29]; even if concerns about the broad use of 4 Gy total body irradiation may recommend some caution until long‐term data are available. We are even more conservative in adult patients, in whom age continues to largely affect long‐term survival together with donor type [30]. Looking at the results of the RACE trial, and in particular to the kinetics of hematological response with triple therapy showing that the vast majority of patients respond within the first 6 or even 3 months (with very low early mortality), we rather argue that in patients for which a clear advantage of a front‐line transplant was not demonstrated (i.e., those lacking a HLA‐identical sibling donor and/or those > 40 years old) initial treatment with triple therapy should be recommended, and transplant (even from mismatched or haplo‐identical donor) [26] may be used as early rescue treatment. While more robust evidence on the role of upfront haplo‐identical transplant may be generated, our recommendation addresses concerns about possible delays in delivering any treatment to patients who may wait months before a transplant is actually performed [31]. On the other hand, we acknowledge that IST (with or without eltrombopag) may remain non‐curative in a substantial proportion of patients due to relapse and/or transformation into myeloid malignancies [17, 19]; thus, treatment decisions may consider all treatment options which may be appropriate in a given patient at a given time, as recently summarized in the American Society of Hematology guidelines on AA [32].

Our study has some limitations. In AA some clinically meaningful event may occur very late in the disease course. Indeed, while disease recurrence tends to occur within months from initial treatment, the emergence of a myeloid malignancy develops after many years, with meaningful cumulative incidence of 10%–15% at 10 years [8, 16, 17]. In our study, “*clonal evolution*” (the standard definition for this event, which is not necessarily accurate) remained very low (three patients in total), with no difference between treatment arms (one vs. two cases). We have also documented by genetic testing that somatic mutations were relatively frequent at baseline, and their prevalence increases over time, with no difference between treatment arms [33]. However, most somatic mutations were not associated with dismal outcome, suggesting that clonal hematopoiesis in aplastic anemia may reflect the expansion of hematopoietic stem cells surviving the immune attack.

However, we have to acknowledge that a longer follow up is needed to see whether the cumulative incidence of myeloid malignancies will increase up to the 15% described in other studies [19]. This long‐term follow up will probably help to tailor treatment for individual patients.

In conclusion, the addition of eltrombopag to the standard ATG and cyclosporine immunosuppressive treatment improves all long‐term outcomes in untreated aplastic anemia patients. While even longer follow up is appropriate to confirm the role of triple therapy in the scenario of disease‐eradicating treatments for SAA, these data prove that eltrombopag on top of standard IST should be the preferred initial therapy for all SAA adult patients not eligible for first‐line hematopoietic stem cell transplantation.

## Author Contributions

Antonio M. Risitano and Régis Peffault de Latour conceived the study, which was designed in collaboration with Simona Iacobelli, Austin Kulasekararaj, Gérard Socié, Ghulam J. Mufti, and Carlo Dufour. Simona Iacobelli made the statistical design, defined the statistical endpoints and the Statistical Analysis Plan, and performed the analyses. All authors gathered the data, vouch for the data and analysis, and for adherence to the protocol. Antonio M. Risitano, Simona Iacobelli, and Régis Peffault de Latour wrote the first draft of the manuscript, which was discussed with Austin Kulasekararaj, Gérard Socié, and Carlo Dufour and then revised by all coauthors. No one who is not an author contributed to writing the manuscript.

## Funding

This study was an EBMT study supported by Novartis, Pfizer, a grant from Alexion Pharma, a grant (A22324) from Cancer Research UK, and grants (10024 and 14017) from Bloodwise UK (previously called Leukaemia and Lymphoma Research).

## Consent

All patients have signed the informed consent approved by local IRB before registration to the trial and starting of all trial procedures.

## Conflicts of Interest

Anna Maria Raiola, Austin Kulasekararaj, Carlo Dufour, and Régis Peffault de Latour have served as members of the advisory board for and have received lecture fees from Novartis and Pfizer. The remaining authors have no conflicts of interest to disclose.

## Supporting information


**Table S1:** Hematological response along follow‐up.
**Table S2:** Cause of death.
**Table S3:** Adverse Events (grade 3 or higher).
**Table S4:** Clonal evolution.
**Table S5:** Self‐reported quality of life (QoL) multivariable models.
**Figure S1:** Response and treatment failure at 2 years.
**Figure S2:** Achievement of first response.
**Figure S3:** Overall Survival (OS) by arm, subgroup analyses according to disease severity and age.
**Figure S4:** Overall Survival (OS) by response, landmark analysis.
**Figure S5:** Event‐Free Survival (EFS), type of failure.
**Figure S6:** Clinical PNH and relapse, Multi Variable Analysis.
**Figure S7:** Cyclosporine discontinuation in responding patients.
**Figure S8:** Additional AA treatments.
**Figure S9:** QoL trajectories derived from multivariable models.

## Data Availability

The data that support the findings of this study are available on request from the corresponding author. The data are not publicly available due to privacy or ethical restrictions.
