# Supplementary material for: Eltrombopag Added to Standard Immunosuppressive Treatment as Front‐Line Therapy for Severe Aplastic Anemia: Long‐Term Outcomes of the Phase‐3 Randomized Superiority EBMT‐SAAWP RACE Study
Source: Am J Hematol. 2026 Jul 19;101(10):2520–32. doi: 10.1002/ajh.70445 (PMC13540314; doi:10.1002/ajh.70445)
Supplement: Supplementary file 1 [file AJH-101-2520-s001.docx]

**Supplemental appendix (index)**

**Study design and Sample Size**

This multicenter prospective randomized clinical trial for SAA patients aimed to compare standard treatment (immunosuppression with hATG + CsA) with an experimental treatment (standard + Eltrombopag). The study adopted a parallel group design, with longitudinal observation of outcomes, and a 1:1 randomization ratio. Randomization was stratified on patient’s age ([15-40) or [40,+∞)), disease severity (Severe (SAA) or Very severe (VSAA) aplastic anemia) and center (grouped according to the expected accrual). The main endpoint of the study was the probability of being in complete response (CR) at month 3 (+/- 10 days). The sample size was determined on the objective of detecting as statistically significant at the 5% level (2-sided test) the superiority of the experimental arm, assuming as expected CR probabilities, respectively, 7% in the standard arm and 21% in the experimental arm. With 96 observations per arm, a test on the difference of these percentages would reach 80% power; the target accrual was thus fixed equal to 200 in total, to account for a possible reduction of randomized individuals (other details available in the study protocol).

**Tables**

**Table S1: Hematological response along follow-up**

**Table S2: Cause of death**

**Table S3: Adverse Events (grade 3 or higher)**

**Table S4: Clonal evolution**

**Table S5: Self-reported quality of life (QoL) multivariable models**

**Figures**

**Figure S1: Response and treatment failure at 2 yrs**

**Figure S2: Achievement of first response**

**Figure S3: Overall Survival (OS) by arm, subgroup analyses according to disease severity and age**

**Figure S4: Overall Survival (OS) by response, landmark analysis**

**Figure S5: Event-Free Survival (EFS), type of failure**

**Figure S6: Clinical PNH and relapse, Multi Variable Analysis**

**Figure S7: Cyclosporine discontinuation in responding patients**

**Figure S8: Additional AA treatments**

**Figure S9: QoL trajectories derived from multivariable models**

**Tables**

| **Table S1: Hematological response along follow-up** | | | | | | | | | |
| --- | --- | --- | --- | --- | --- | --- | --- | --- | --- |
| **Response** | **3 Months** | | | | **6 Months** | | | **24 Months** | |
|  | **hATG+CsA**  **(Arm A)**  **(N=101)** | **hATG+CsA+EPAG**  **(Arm B)**  **(N=96)** |  | **hATG+CsA**  **(Arm A)**  **(N=101)** | | **hATG+CsA+EPAG**  **(Arm B)**  **(N=95)** | **hATG+CsA**  **(Arm A)**  **(N=99)** | | **hATG+CsA+EPAG**  **(Arm B)**  **(N=93)** |
|  | *no. of patient. (%)* | |  | *no. of patients (%)* | | | *no. of patients (%)* | | |
| No response | 70 (69·3%) | 39 (40·6%) |  | 60 (59·4%) | | 31 (32·6%) | 63 (63·6%) | | 44 (47·3%) |
| Partial response | 21 (20·8%) | 36 (37·5%) |  | 21 (20·8%) | | 34 (35·8%) | 6 (6·1%) | | 10 (10·8%) |
| Complete response | 10 (9·9%) | 21 (21·9%) |  | 20 (19·8%) | | 30 (31·6%) | 30 (30·3%) | | 39 (41·9%) |
|  |  |  |  |  | |  |  | |  |
| Overall response† | 31 (30·7%) | 57 (59·4%) |  | 41 (40·6%) | | 64 (67·4%) | 36 (36·4%) | | 49 (52·7%) |
|  |  |  |  |  | |  |  | |  |
|  | *Arm B vs Arm A:* | |  | *Arm B vs Arm A:* | | | *Arm B vs Arm A:* | | |
|  | *OR (95% CI)** | *P Value* |  | *OR (95% CI)** | | *P Value* | *OR (95% CI)** | | *P Value* |
| Complete response | 3·2 (1·3-7·8) | 0·012 |  | 2·3 (1·1-4·7) | | 0·019 | 1·94 (1·04-3·63) | | 0·037 |
| Overall response | 4·2 (2·2-8·1) | <0·001 |  | 3·5 (1·9-6·5) | | <0·001 | 2·42 (1·31-4·48) | | 0·005 |

Abbreviations: CsA, cyclosporine; EPAG, eltrombopag; GPI, glycophosphatidylinositol; hATG, horse antithymocyte globulin; no., number

No response includes cases who, before the assessment time, were deceased, or experienced clonal evolution, or received transplantation or additional AA treatment. No Response at 24 months (last visit) was defined based on the same criteria, adding cases who relapsed.

†Overall response includes partial or complete response.

Cases not evaluated (1 at 6 months, 5 at 24 months) withdrew consent to further data collection before the assessment. One case was reclassified at 6 months based on additional information on treatments received, acquired after the first publication (arm B, from PR to NR).

*Mantel-Haenszel pooled Odds Ratio (OR) B vs A (with 95% Confidence Interval) stratified by Age, Severity, and Centre.

| **Table S2: Cause of death** | | | |
| --- | --- | --- | --- |
|  | **Arm A** | **Arm B** | **Total (%)** |
| Infections | 9 (64%) | 4 (50%) | 13 (59%) |
| Bleeding | 2 (14%) |  | 2 (9%) |
| Other | 3 (21%) | 4 (50%) | 7 (32%) |
| *Details:* |  |  |  |
| Lung cancer | 1 |  |  |
| Encephalopathy of unknown origin | 1 |  |  |
| Acute respiratory distress syndrome of unknown origin |  | 1 |  |
| Aortic valve disease |  | 1 |  |
| Cardiac tamponade |  | 1 |  |
| Thrombosis |  | 1 |  |
| Transplant-related mortality | 1 |  |  |
| **Total** | **14** | **8** | **22** |

| **Table S3: Adverse Events (grade 3 or higher)** | | | |
| --- | --- | --- | --- |
| **SOC** | **hATG+CsA**  **(Arm A)**  **(N=101)** | **hATG+CsA+EPAG**  **(Arm B)**  **(N=96)** | **Total**  **(N=197)** |
| Blood and lymphatic system disorders | 38 | 33 | 71 |
| Cardiac disorders | 7 | 6 | 13 |
| Ear and labyrinth disorders | 2 | 0 | 2 |
| Endocrine disorders | 0 | 1 | 1 |
| Eye disorders | 1 | 2 | 3 |
| Gastrointestinal disorders | 8 | 24 | 32 |
| General disorders and admin. site conditions | 19 | 21 | 40 |
| Hepatobiliary disorders | 17 | 7 | 24 |
| Immune system disorders | 10 | 4 | 14 |
| Infections and infestations | 77 | 61 | 138 |
| Investigations | 30 | 20 | 50 |
| Metabolism and nutrition disorders | 14 | 6 | 20 |
| Musculoskeletal and connective tissue disorders | 8 | 6 | 14 |
| Neoplasms benign, malignant and unspecified | 6 | 1 | 7 |
| Nervous system disorders | 8 | 9 | 17 |
| Psychiatric disorders | 3 | 1 | 4 |
| Renal and urinary disorders | 10 | 6 | 16 |
| Respiratory, thoracic and mediastinal disorders | 8 | 9 | 17 |
| Skin and subcutaneous tissue disorders | 9 | 5 | 14 |
| Social circumstances | 1 | 0 | 1 |
| Surgical and medical procedures | 4 | 5 | 9 |
| Vascular disorders | 14 | 10 | 24 |
| Unclassified | 0 | 1 | 1 |
| Total | 294 | 238 | 532 |

**Table S4: Clonal evolution**

| **Arm** | **Age** | **Severity** | **Response** | | **CE** | **Relapse** | **Transplant** | **Other Additional AA Treatment (start)** | **Status 24 mo.** | **Karyotypic abnormalities** | | | **Somatic mutations (VAF)** | | |
| --- | --- | --- | --- | --- | --- | --- | --- | --- | --- | --- | --- | --- | --- | --- | --- |
|  |  |  | **3 mo.** | **6 mo.** |  |  |  |  |  | **Baseline** | **6 months** | **24 months** | **Baseline** | **6 months** | **24 months** |
| A | 58 | Severe | PR | NR | 5.6mo | 10.6mo | 13.2mo | None | Alive 23.3mo | 46XY* | 46, XY, +Y, -7[4]/  46, XY[11] | N.A. | None | TET2 (13.2%)  DNMT3A (13.2%)  BCOR (7.7%; 6.3%) | Not Available |
| B | 62 | Severe | NR | NR | 6.2mo | No | No | Eltrombopag (12.3mo) | Alive 23.9mo, PR | 46,XY [15] | 46,XY,-13(q13q34)[2]/  46,XY[18] | Unknown  *(persistent del13q at 12 and 18 months)* | None |  | Not Available |
| B | 19 | Severe | PR | CR | 12.0mo | No | No | None | Alive 23.7mo, CR (CsA discontinued) | 46,XX[16] | Not done or failed  *(del13q at 12 &18 months)* | Normal | PIGA (7.7%; 3.2%; 2.4%) | PIGA (6.6%) | Not Available |

Information for the 3 cases who experienced Clonal Evolution (CE); none showed morphological signs of MDS. CsA: cyclosporine A; N.A.=not applicable; mo=months.

*Number of metaphases not available/not reported by center

| **Table S5: Self-reported quality of life (QoL) multivariable models** | | |
| --- | --- | --- |
|  | **QL2** | **FA** |
| **(Intercept)** | 56.04** | 43.68** |
| **Arm†** | 3.22 | 0.25 |
| **Time 6 months** | 13.08** | -11.29** |
| **Time 12 months** | 20.62** | -16.32** |
| **Time 24 months** | 19.53** | -15.29** |
| **Arm: time 6 months** | -1.93 | 2.87 |
| **Arm: time 12 months** | -3.34 | 3.34 |
| **Arm: time 24 months** | 0.68 | -0.01 |
| **Age†** | -5.52* | -0.10 |
| **Disease severity†** | -4.11 | -0.32 |

Abbreviations: QL2, Global health status (higher = better); FA, fatigue scale (higher =worse); †Arm, randomization arm (B vs A); Age, ≥40 vs ≥15 and <40; Disease severity, very severe aplastic anemia (vSAA) versus severe aplastic anemia (SAA).

Results of the linear mixed models for the quality of life scales: regression coefficients with significance, ** p<0.01; * p<0.05; ° p<0.1. The models evaluate the effect of time (categorical: t=0 months baseline, t=6 months, 12 months, 24 months), the effect of randomization arm (B vs A), and of their combination, thus assessing whether the trend in time was different according to arm. All models include also age (≥40 vs ≥15 and <40) and Disease Severity (vSAA vs SAA) as adjustment factors, plus a random intercept for the “patient effect” and an adjustment for centre effect (not shown). Both scales showed an improvement of QoL in time. There was no difference of the trend between arms.

Relevant trajectories derived from these models are illustrated in **Figure S9**.

**Figures**

**Figure S1: Response and treatment failure at 2 yrs**

A: Hematological response status (Complete Response or Partial Response) observed at 2 years in absence of prior treatment failure, by arm; stacked proportions. B: Type of treatment failure, by arm; proportions. Failure was the first event of the following, occurring before the evaluation at 2 years: death, relapse (Rel), stem cell transplantation (SCT), additional AA treatment (AT).

**Figure S2: Achievement of first response**

A: Achievement of first Complete Remission (CR). B: Achievement of first overall response. Cumulative incidence curves, by arm. Competing events: death, transplant, myeloid malignancy, additional AA treatment.

**Figure S3: Overall Survival (OS) by arm, subgroup analyses according to disease severity and age**

Overall Survival (OS) by arm, subgroup analyses according to disease severity and age. Kaplna-Meier curves. A: Severe AA patients; B: Very severe AA patients; C: Patients <=40yo; D: Patients >40yo

**Figure S4: Overall Survival (OS) by response, landmark analysis**

Kaplan-Meier curves, conditional on survival at 3 and 6 months respectively. A: Comparison by response status at 3 months. B: Comparison by response status at 6 months.

A	B

**Figure S5: Event-Free Survival (EFS), type of failure**

Event-Free Survival (EFS), type of failure. Stacked cumulative incidence curves for each type of failure. Failure was the first event of the following: death, no response at 6 months, myeloid malignancy, relapse, stem cell transplantation, additional AA treatment. A: Arm A; B: Arm B

**Figure S6: Clinical PNH and relapse, Multi Variable Analysis**

Multi-variable analysis, forest plot (HR with 95%CI). A: Clinical PNH; B: Relapse. C. Cumulative incidence of relapse, estimated from time of first hematological response.

**C.**

**Figure S7: Cyclosporine discontinuation in responding patients.**

A. Stacked curve of cyclosporine discontinuation in Arm A. B. Stacked curve of cyclosporine discontinuation in Arm B. A and B: in purple competing events (death, clonal evolution, new treatments, transplant), in green immunosuppressive discontinuation (ISD)

**A.**

**B.**

**Figure S8: Additional AA treatments.**

Number of additional AA treatments per patient, by arm; proportions.

**Figure S9: QoL trajectories derived from multivariable models**

Visualization of the results of the QoL multivariable models (**Table S5**). Longitudinal trajectories of: A: QL2, Global health status (higher = better); B: FA, fatigue scale (higher = worse).

A.

B.
